# Intra-individual changes in haemosporidian infections over the nesting period in great tit females

**DOI:** 10.1007/s00436-017-5540-9

**Published:** 2017-07-01

**Authors:** Anna Dubiec, Edyta Podmokła, Lars Gustafsson

**Affiliations:** 1 Museum and Institute of Zoology, Polish Academy of Sciences, Warszawa, Poland; 2 Institute of Environmental Sciences, Jagiellonian University, Kraków, Poland; 3 Department of Ecology and Genetics, Evolutionary Biology Centre, Uppsala University, Uppsala, Sweden

**Keywords:** Haemosporidians, Individual quality, Mixed infections, Resident host, Seasonal change

## Abstract

Prevalence of haemosporidian parasites in bird populations varies temporally both between years and within a year. In contrast to variation at the population level, relatively little is known about variation in infection attributes at the individual level, especially in non-migratory species. We examined intra-individual changes in the presence and identity of haemosporidian parasites (genera *Plasmodium* and *Haemoproteus*) over the course of the nesting period in females of great tits (*Parus major*)—a species considered to be resident over much of its distribution range. Birds were sampled during two stages of the nesting period: nest building and nestling rearing. The mean time interval between sampling occasions was 43 days. Between the first and second samplings, 30.6% of females gained at least one parasite lineage and 18.5% lost the lineage. *Haemoproteus* gains were over three times more common than *Plasmodium* gains. The probability of the lineage gain decreased with the date of the first sampling, was higher in individuals in better body condition and differed between years, but was not associated with the host age. The probability of the lineage loss was not explained by any of the considered parameters except for year. These results indicate that in a large proportion of a population, infection attributes (presence/absence and/or parasite identity) may change over the nesting period and the occurrence of such changes may be associated with the individual quality. Consequently, this phenomenon should be taken into account to correctly interpret parasite-mediated effects.

## Introduction

Haemosporidian protoza from the genera *Plasmodium* and *Haemoproteus* are one of the commonest parasites in birds (Valkiūnas 2005). Prevalence of these vector-borne parasites is not only very variable among host species, but also varies among populations of the same host species and within the same population (Bensch and Åkesson 2003; Pagenkopp et al. 2008; Szöllősi et al. 2011). In this latter case, prevalence has been shown to vary between years as well as seasonally (Schrader et al. 2003; Bensch et al. 2007; Cosgrove et al. 2008; Deviche et al. 2010).

While between- and within-year variation in haemosporidian infections at the population level is currently rather well explored, relatively little is known about variation in infection at the individual level. Importantly, most available information on intra-individual patterns refers to between-year variation (e.g. Bensch et al. 2007; Hasselquist et al. 2007; van Rooyen et al. 2013; Synek et al. 2013; Podmokła et al. 2017). These data indicate that host species vary in the observed patterns. While in some species, the majority of individuals retains the infection status or parasite identity between successive samplings (Hasselquist et al. 2007; van Rooyen et al. 2013; Hammers et al. 2016), in others most individuals change infection status and/or parasite type between years (Piersma and van der Velde 2012).

Available information about intra-individual seasonal changes in infection status mostly refers to long and intermediate distance migrants. Because of differences in physiology associated with migratory behaviour and exposure to a more diverse parasite community (on breeding and wintering grounds and at stopover sites) (Waldenström et al. 2002; Garvin et al. 2006; Hellgren et al. 2007), migratory and non-migratory birds may differ with respect to patterns of within-seasonal changes in haemosporidian infections. Current data indicate that in migrants most individuals retain the infection status over the course of the breeding period and when the change occurs, it is dominated by disappearance of infection from the peripheral blood (Dale et al. 1996; Hasselquist et al. 2007; Piersma and van der Velde 2012; Szöllősi et al. 2016, but see Marzal et al. 2005 for a predominance of infection gains). The few studies focusing on non-migratory species suggest the existence of a similar pattern, namely that most individuals retain the infection status with the progress of the nesting period, and in the group of birds transitioning between infection status, infection loss is more common than its gain, although this pattern is not necessarily followed by all parasite species (Merilä and Andersson 1999; Knowles et al. 2011). For example, Knowles et al. (2011) reported that in blue tits sampled at the beginning and the end of the 2-week period spanning the nestling stage nearly 20% of females lost infections of *Plasmodium relictum*, while the loss of *Plasmodium circumflexum* was negligible. At the same time, infection gains were low and did not differ between parasite species. However, it is difficult to predict whether such temporal pattern of haemosporidian infections may be considered universal in temperate non-migratory birds because of a very limited number of studies. Given physiological and behavioural differences between host species, parasite species/lineages predominantly infecting a given population and the degree of an overlap of the host breeding period and the activity of the vector community, differences in this pattern are highly possible.

Information about the frequency and the type of intra-individual changes in infection status over the course of the breeding period is crucial for a correct interpretation of parasite-mediated effects. For example, in many studies focusing on the consequences of haemosporidian infections for reproduction, adult birds are sampled once, at the late stage of the nesting period, i.e. several days before the nestlings fledge (e.g. Marzal et al. 2008; Knowles et al. 2011; Podmokła et al. 2014b). If, however, the high proportion of birds in the population changes infection status or the parasite species/lineage over the course of the nesting period, using infection parameters acquired at the end of the nesting period, may obscure some associations or lead to false conclusions. This should be especially true for linking late infection status with reproductive performance at the early nesting stages, e.g. investment in nest construction, egg production or incubation.

In the current study, we examined changes in the presence and identity of haemosporidian parasites over the course of the nesting period in individual great tits (*Parus major*). Great tit is a small cavity-nesting passerine, which is considered resident over much of southern and central part of its distribution range and irregular eruptive or regular partial migrant in northern and north-eastern areas (Cramp and Perrins 1993; Nowakowski and Vähätalo 2003). The study was conducted in a Swedish population characterized by a high incidence of infection with *Plasmodium*/*Haemoproteus* parasites (Dubiec et al. 2016). Birds were sampled during two stages of the nesting period: nest building and nestling rearing. Sampling was limited to females because males are more difficult to catch at the early stages of the nesting period. We assessed the frequency of parasite lineage gains and losses over the nesting period and investigated whether the occurrence of these changes was associated with the date of initial sampling, body condition and age of the bird. Date of sampling may associate with changes in infection attributes because of the seasonal variation in the occurrence of insects vectoring haemosporidian parasites and temporal patterns of parasite occurrence in the peripheral blood associated with a relapse of latent infections (Applegate 1970; Sundberg 1995; Ander et al. 2012). Body condition may play a role in this process through its influence on immune function and the corticosterone level (Kitaysky et al. 1999; Møller and Petrie 2002), while age is known to relate to the prevalence and intensity of haemosporidian infections with yearlings having less prevalent and more intense infections than older birds (Allander and Bennett 1994; Allander and Sundberg 1997).

## Methods

Data were collected during two breeding seasons (2011–2012) in a nest box breeding population of great tits in southern Gotland, Sweden (57° 03′ N, 18° 17′ E) as part of the study focusing on the consequences of haemosporidian infections for reproductive output (Dubiec et al., in prep). The study site consists of over 10 large and several small wood plots (primarily deciduous) separated by arable areas (for the map of the study area, see Dubiec et al. 2016). Data used in this study were collected in 7 plots in 2011 and 8 plots in 2012.

From the middle of April nest boxes were checked every second day to locate boxes, in which tits started nest building. When material indicating tit nest was found, bird trapping started the following day(s). At this stage of the nesting period, birds were caught with traps installed inside the box. Trapping was continued until the female was caught or until the first egg appeared in the nest, when it was ceased. Most females were trapped when the nest was half-built (the nest box floor was covered with a thick layer of moss, but a cup was not present). Because in great tits nest is built by female alone (Cramp and Perrins 1993) and therefore they are much easier to catch than males at this stage of the nesting period, only females were used in this study. Caught birds were sampled for blood, ringed (if not already ringed) with an aluminium ring, sexed, aged and weighed. Birds were sexed based on the width and the degree of gloss of the stripe of black feathers on the belly and aged as yearlings or at least 2 years old based on plumage characteristics (Svensson 1994). In the case of birds ringed in previous years, age was later verified using ringing records. For the purpose of the study investigating the effects of haemosporidian infections on reproductive output, females were assigned in turns to two treatment groups: (i) control, which received the intraperitoneal injection of 0.1 ml of a physiological salt solution (0.9% NaCl) and (ii) medicated, which received injection of an anti-malarial drug—primaquine (Sigma-Aldrich) in a dose of 0.01 mg in 0.1 ml of a physiological salt solution (Merino et al. 2000). Because primaquine may potentially eradicate malaria parasites and influence the probability of developing a new infection, females subject to this treatment and later re-caught during the nestling stage were excluded from the analyses. All females except for five deserted the nest box after the capture. Because empty nest boxes were visited until no new nests were found, some females were caught more than once (7 females—twice, 1 female—three times). On the second and following captures, females were immediately released after the identification based on the ring number (except for 1 female, which escaped during the first capture and was sampled and injected with a physiological salt solution during the second capture after 4 days).

Nests were visited throughout the nesting period regularly to record the set of breeding parameters (data not shown). Starting from day 9 post-hatching (hatching day = day 0), adult birds were caught (modal nestling age at catching of adult birds—14 days) either with a nest box trap or a mist net set in the vicinity of the box. Birds were sampled for blood, weighed (to the nearest 0.1 g) and had a set of morphometric measures taken (including among others tarsus length and wing length, to the nearest 0.1 mm). During both captures blood samples were obtained from the wing vein (~30–50 μl) using non-heparinized capillaries and stored in 96% ethanol in ambient temperature until molecular analyses.

### Molecular analyses of infection status and identification of haemosporidian lineages

Genomic DNA was extracted from the blood using an ammonium acetate method (Bruford et al. 1998). Samples were screened for the presence of blood parasites (genus *Haemoproteus* and *Plasmodium*) by amplifying a 478-bp fragment of the mitochondrial cyt *b* gene, using nested polymerase chain reaction (Waldenström et al. 2004). PCR conditions followed the protocol of Cosgrove et al. (2008) and PCR products were processed as described in Podmokła et al. (2014a, 2014b). The parasite lineages were identified as described in Dubiec et al. (2016). In short, sequences were visually inspected and identified by aligning to sequences in the MalAvi database (Bensch et al. 2009). In the case of mixed infections, i.e. infections caused by two or more lineages simultaneously (indicated by multiple peaks in the chromatogram), parasite lineages were assigned following the visual comparison of sequences with the pool of lineages known to occur at the study site or when it was impossible—by cloning of the PCR product.

### Body condition index

As an estimate of body condition we used the scaled mass index (calculated separately for each year) following Peig and Green (2009). This index is a better indicator of the relative size of energy reserves and other body components than residuals from an ordinary least squares regression of body mass against a linear morphometric measure representing size, because the latter index has been shown to be biased towards larger individuals (Peig and Green 2009, 2010). In principle, this method scales the mass of an individual to that expected if all individuals in the sample had the same structural size. As a measure of structural size we used tarsus length, because it showed higher correlation with body mass than other measured morphometric characters (*r* = 0.49 and 0.52 in 2011 and 2012, respectively).

### Sample size and statistical analyses

In total, in two breeding seasons, there were 71 females caught during the nest building stage which received injection with a physiological salt solution. Additionally, in 2012, there was 1 female which was sampled for blood, but escaped before receiving the injection. Fifty-one of these 72 females (2011—18, 2012—33) were caught at the late nesting stage. Datasets from two females were excluded from statistical analyses because either the blood sample was not acquired during the second capture or the female was sampled in both breeding seasons (in the latter case only data from the first season were used). Consequently, 49 data records from unique females were available for statistical analyses.

Temporal changes in haemosporidian infections at the individual level were examined using generalized linear models (GLM) with binomial error structure and a logit link function. As a dependent variable we used: (1) the probability of the lineage(s) gain (coded as 1 when at least one lineage was acquired between the first and second samplings in either uninfected or infected females and 0 in all other cases) and (2) the probability of the lineage loss (coded as 1 when at least one lineage was lost between the first and second samplings in infected females and 0 in all other cases). In the latter model, only females which were infected during the first sampling were included in the analyses. Five females were coded as 1 in both models because they experienced the lineage acquisition as well as the lineage loss between sampling occasions. Explanatory variables included the following: year, date of the first sampling, female age and body condition. Sample size in GLM models was reduced by 3 females, which were not weighed during the first sampling. The analyses were conducted with IBM SPSS ver. 19 (IBM Corp. 2010).

## Results

During the nest building stage the median caching date was 24 April in 2011 (range 19 April–4 May) and 25 April in 2012 (21 April–1 May). In both years, mean time interval between the date the female was sampled for blood and started egg laying was 7 ± 3 (SD) days (range 2011—2–12 days, 2012—3–15 days). During the nestling period, the median catching date was 6 June in 2011 (range 29 May–16 June) and 9 June in 2012 (range 31 May–14 June). The mean time interval between sampling occasions was 43.8 ± 2.4 days in 2011 (range from 40 to 49 days) and 43.2 ± 3.8 days (range from 34 to 51 days) in 2012.

On average 88.7% (2011, 100%; 2012, 77.4%) of females carried the haemosporidian infection when caught during the nest building stage (*n* = 49). The list of parasite lineages and the frequency of their occurrence during the first sampling is presented in Table 1. 11.5% of females (2011, 16.7%; 2012, 6.4%) carried mixed infections, all composed of two lineages. Between the first and second sampling occasions, 38.8% of females changed infection attributes including 30.6% which gained and 18.5% which lost the lineage. In the case of lineage gains in 33.3% of females more than one lineage was acquired by the time of the second sampling, while in the case of losses in all individuals only one lineage disappeared. Because the loss of the lineage in all cases occurred in individuals carrying a mixed infection or in individuals, in which it was accompanied by the acquisition of a new lineage, there was not a single case of the transition from an infected to uninfected status. *Haemoproteus* gains were over three times more common than *Plasmodium* gains (86.7 vs 26.7%, Fisher exact test, *p* < 0.01). The list of lineages which were acquired between the first and second samplings and the frequency of each lineage gain are presented in Table 2.

**Table 1:** The community of *Plasmodium* and *Haemoproteus* parasites in great tit females sampled during the nest building stage on Gotland (Sweden) in years 2011–2012

Parasite lineage	Parasite taxon	GenBank accession no.	2011 *n* = 18	2012 *n* = 31
BT7	*Plasmodium circumflexum*	AY393793	1 (5.6)	1 (3.2)
GRW11	*Plasmodium relictum*	AY831748	1 (5.6)	1 (3.2)
PARUS66	*Plasmodium* sp.	KU695263	1 (5.6)	0 (0.0)
SGS1	*Plasmodium relictum*	AF495571	0 (0.0)	2 (6.5)
SW2	*Plasmodium homonucleophilum*	AF495572	5 (27.8)	2 (6.5)
TURDUS1	*Plasmodium circumflexum*	AF495576	11 (61.1)	13 (41.9)
PARUS1	*Haemoproteus majoris*	AF254977	1 (5.6)	4 (12.9)
PARUS65	*Haemoproteus* sp.	KU695262	0 (0.0)	1 (3.2)
PHSIB1	*Haemoproteus majoris*	AF495565	1 (5.6)	2 (6.5)

The number and prevalence (in brackets) of each parasite lineage is given separately for each breeding season. Prevalence of each lineage was calculated using individuals with single and mixed infections

*N* the number of screened individuals

**Table 2:** The list of *Plasmodium* and *Haemoproteus* lineages which were acquired between sampling during the nest building and nestling stage in great tit females (*n* = 15). In 6 individuals more than one lineage was acquired

Parasite lineage	No. of individuals (%)
BT7	1 (6.7)
SGS1	2 (13.3)
SW2	2 (13.3)
WW2^a^	2 (13.3)
PARUS1	6 (40.0)
PHSIB1	9 (60.0)

^a^Lineage WW2 represents *Haemoproteus majoris* (GenBank accession no.: AY831755)

The probability of the lineage gain decreased with the date of first sampling, increased with the scaled mass index and was higher in 2012 (Table 3). Age did not explain the probability of acquiring new parasite lineage(s) between early and late nesting period. In the case of the lineage loss, only year explained variation in the incidence of this type of infection change (Table 3).

**Table 3:** Results of generalized linear models predicting the acquisition or loss of the *Plasmodium*/*Haemoproteus* lineage(s) in great tit females between nest building and nestling rearing stages

Dependent variable (*n*)	Explanatory variables	df	Parameter estimate ± SE	LR *χ* ^2^	*p* value
Lineage acquisition (46)	Year (2011)	1	4.18 ± 1.64	12.39	<0.001
	Date of the first sampling	1	−0.43 ± 0.20	6.04	0.014
	Age (yearling)	1	0.23 ± 0.93	0.06	0.801
	Scaled mass index	1	1.40 ± 0.54	11.46	0.001
Lineage loss (39)	Year (2011)	1	1.89 ± 1.00	4.29	0.038
	Date of the first sampling	1	−0.14 ± 0.14	0.97	0.325
	Age (yearling)	1	−1.11 ± 1.00	1.39	0.239
	Scaled mass index	1	0.12 ± 0.35	0.13	0.722

In the case of categorical explanatory variables, the reference level of the variable is given in brackets

*N* the number of individuals

## Discussion

Our study shows that a large proportion of the population (nearly 40%) of a predominantly non-migratory bird species changes the haemosporidian infection status at the individual level over the course of the nesting period. The main type of the change was the acquisition of the haemosporidian lineage(s). Nearly 87% of lineages gained between sampling occasions belong to *Haemoproteus* genus.

The findings of our study partly contrast with patterns found in other studies examining intra-individual changes in the haemosporidians infection status over the breeding season, both in migratory and non-migratory species. While similarly to other studies, the majority of great tit females retained the infection status over the nesting period, among birds which did change it, the majority experienced the acquisition of the new lineage(s). None of the females completely cleared the infection from the peripheral blood, although some females carrying a mixed infection lost the lineage or the lineage was replaced by a new one(s). In contrast, in the majority of other studies the predominant type of infection change was the loss of parasites from the peripheral blood (Knowles et al. 2011; Piersma and van der Velde 2012; Szöllősi et al. 2016, but see Grillo et al. 2012). For example, in a long-distance migrant, collared flycatchers (*Ficedula albicollis*), 76.5% of males infected with *H. pallidus* during courtship lost this infection by the nestling rearing period (Szöllősi et al. 2016), while in non-migratory blue tits ca 20% of females lost *Plasmodium* infections over the 2-week period spanning the nestling stage (Knowles et al. 2011).

Based on data collected in this study, it is not possible to definitely resolve whether the acquisition of lineages occurred as a result of a relapse of latent infections or new transmissions. In temperate regions, in many avian host species, *Plasmodium* and *Haemoproteus* parasites disappear from the peripheral blood during some winter months and after a period of a dormant stage in tissues of different organs reappear in circulating red blood cells. This phenomenon, known as “a spring relapse”, has been shown to be triggered by photoperiod and stress, probably via changes in the level of gonadal hormones and corticosterone (Applegate 1970; Applegate and Beaudoin 1970; Deviche et al. 2001; Valkiūnas et al. 2004). The acquisition of parasites via new transmission requires the presence of competent vectors: mosquitoes (Culicidae) in the case of *Plasmodium* and biting midges (Ceratopogonidae) and louse flies (Hippoboscidae) in the case of *Haemoproteus* (Valkiūnas 2005). Currently, there is little data on the occurrence and phenology of ornithophilic dipterans vectoring haemosporidians in the study area. Two ornithophilic mosquito species (*Culex pipiens* and *Culex torrentium*) and at least five ornithophilic species of *Culicoides* biting midges has been detected so far on Gotland (Ander et al. 2012; Lundström et al. 2013). However, their phenology in the study area has not been described yet. In general, first biting midges occur on Gotland in the second week of May (Ander et al. 2012) and first adult mosquitoes in the middle of May (own observations), although as occurrence of dipteran vectors is dependent on weather conditions it may vary between years. Since the development of sporozoites in vectors takes 4–8 days in the case of *Haemoproteus* and a week in the case of *Plasmodium* and a prepatent period in the vertebrate host may be as short as several days, it may not be excluded that some birds sampled in June carried newly transmitted haemosporidian infections (Fallis and Bennett 1960; Valkiūnas 2005). Higher probability of the parasite gain in females caught earlier in the season strongly indicates that the relapse is mostly responsible for appearance of parasites in the blood. The opposite pattern is expected if new transmissions contribute to parasite gains as a consequence of the seasonal pattern of dipteran vectors’ occurrence. Other indication for a relapse as a main source of lineage acquisition is a positive association between the probability of the lineage gain and female body condition. This finding may suggest that females having larger reserves of energy and other body components may delay the transition of parasites from tissue-only forms to stages present in the blood.

Higher proportion of *Haemoproteus* among acquired lineages may be explained by at least three mechanisms. Firstly, in temperate regions *Haemoproteus* infections may on average relapse later than *Plasmodium* infections. If this is the case, most great tit females sampled in the second half of April would already carry relapsed *Plasmodium* infections, and only some—*Haemoproteus* infections. For example, in the yellowhammer, prevalence of *Haemoproteus* infections steeply increases, reaching over 90%, in the last third of April with less than 50% of individuals being infected in March and in the first and second third of April (Sundberg 1995). While, to our knowledge, there are no studies corroborating the prediction of differential timing of *Plasmodium* and *Haemoproteus* relapses, available data suggests the existence of such difference for example between *Haemoproteus* and *Leucocytozoon*, with the former genus relapsing later in the season (Deviche et al. 2010). Secondly, *Plasmodium* and *Haemoproteus* vectors may differ in the timing of occurrence and/or the degree of endophagy (attacking the host in enclosed places). While the direct comparison of phenology of ornithophilic haemosporidian vectors in the study area is lacking, available data and own observations indicate that they may emerge at a similar time of the season (Ander et al. 2012). Even if this is the case, vectors of *Haemoproteus* seem to show higher degree of endophagy. Votýpka et al. (2009) recorded in central Czech over 35 times more *Culicoides* than Culicidae specimens in nest boxes (using sticky foil attached to the upper part of the nest box as an insect collection method) occupied by small passerines. Higher endophagy of *Hemoproteus* vectors should in turn translate into higher transmission rates of these parasites in great tit females since in this species only female incubates and broods the young and consequently spends most of the day in the nest box. Thirdly, the reported pattern may be associated with the properties of used molecular technique. While molecular screening based on nested PCR has been shown to have higher sensitivity than traditionally used blood smear diagnostics (Richard et al. 2002; Fallon et al. 2003; Garamszegi 2010, but see Valkiūnas et al. 2006; Valkiūnas et al. 2008 for comparable estimates), it is known to miss some infections. It has been demonstrated that the probability of infection detection using nested PCR increases with increasing infection parasitaemia (Knowles et al. 2011). Because parasitaemia of relapsed *Haemoproteus* infections is initially low (Allander and Sundberg 1997), birds sampled early in the season may be scored as uninfected simply because nested PCR is not sensitive enough to detect such infections. Moreover, nested PCR does not detect all cases of mixed infections, which may be associated with preferential amplification of lineages with higher parasitaemia (Pérez-Tris and Bensch 2005; Valkiūnas et al. 2006; Bernotienė et al. 2016).

In conclusion, we showed that in a predominantly non-migratory bird species the incidence of change in the haemosporidian infection status over the nesting period is high. Contrary to the majority of previous studies, we found that the main type of infection change was the acquisition of the lineage(s). These findings indicate that the phenomenon of intra-individual within-seasonal changes in infection attributes should be taken into account in order to correctly interpret parasite-mediated effects. However, more studies, especially in non-migratory species, are needed to assess how common are different patterns of intra-individual changes in infection attributes across bird species and populations.
